# Supplementary material for: Melanoblast transcriptome analysis reveals pathways promoting melanoma metastasis
Source: Nat Commun. 2020 Jan 16;11:333. doi: 10.1038/s41467-019-14085-2 (PMC6965108; doi:10.1038/s41467-019-14085-2)
Supplement: Supplementary file 1 — Supplementary Information [file 41467_2019_14085_MOESM1_ESM.pdf]

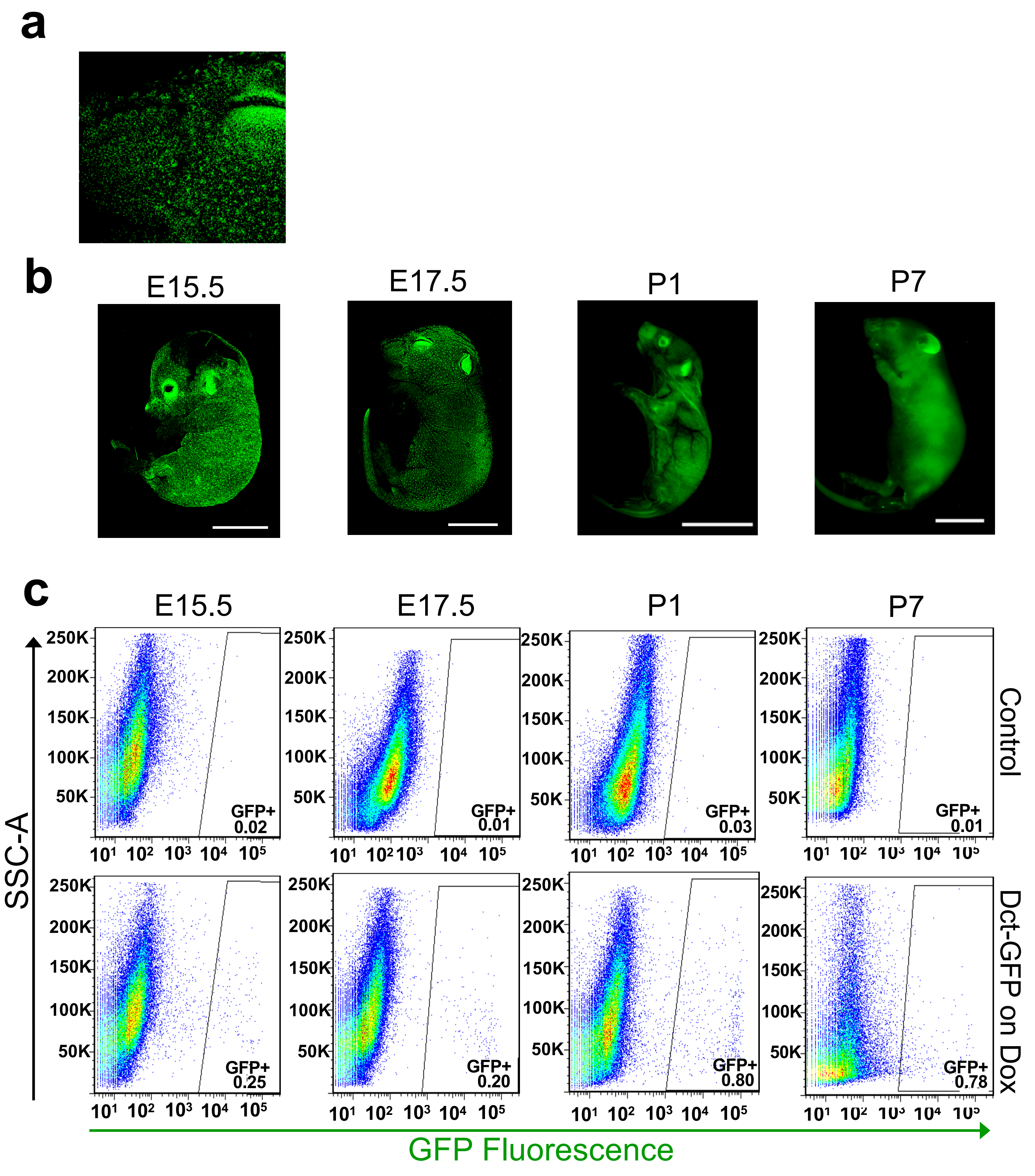
a
b
E15.5
E17.5
P1
P7
c
E15.5
E17.5
P1
P7
SSC-A
250K
200K
150K
100K
50K
GFP+ 0.02
GFP+ 0.01
GFP+ 0.03
GFP+ 0.01
GFP+ 0.25
GFP+ 0.20
GFP+ 0.80
GFP+ 0.78
Control
Dct-GFP on Dox
10^1 10^2 10^3 10^4 10^5
GFP Fluorescence

Supplementary Figure 1: Characterizing the mouse melanoblast transcriptome. a-b, Confocal imaging of i*Dct*-GFP embryos and pups. Zoom in of Embryonic day 17.5 pup showing melanoblasts localized to hair follicles on face (**a**). Embryonic day 15.5 and 17.5 (E15.5 and E17.5 respectively; **b**). Postnatal day 1 and postnatal day 7 (P1, P7 respectively; **b**). Magnification E15.5 and E17.5 is x5, scale bars, 5 mm; magnification P1 and P7 is x1. Scale bars, 10 mm. **c**, FACS-sorting of GFP-positive cells from i*Dct*-GFP mice. SSC-A: Side scatter area. Proportion of GFP-positive cells indicated (GFP^+).

a

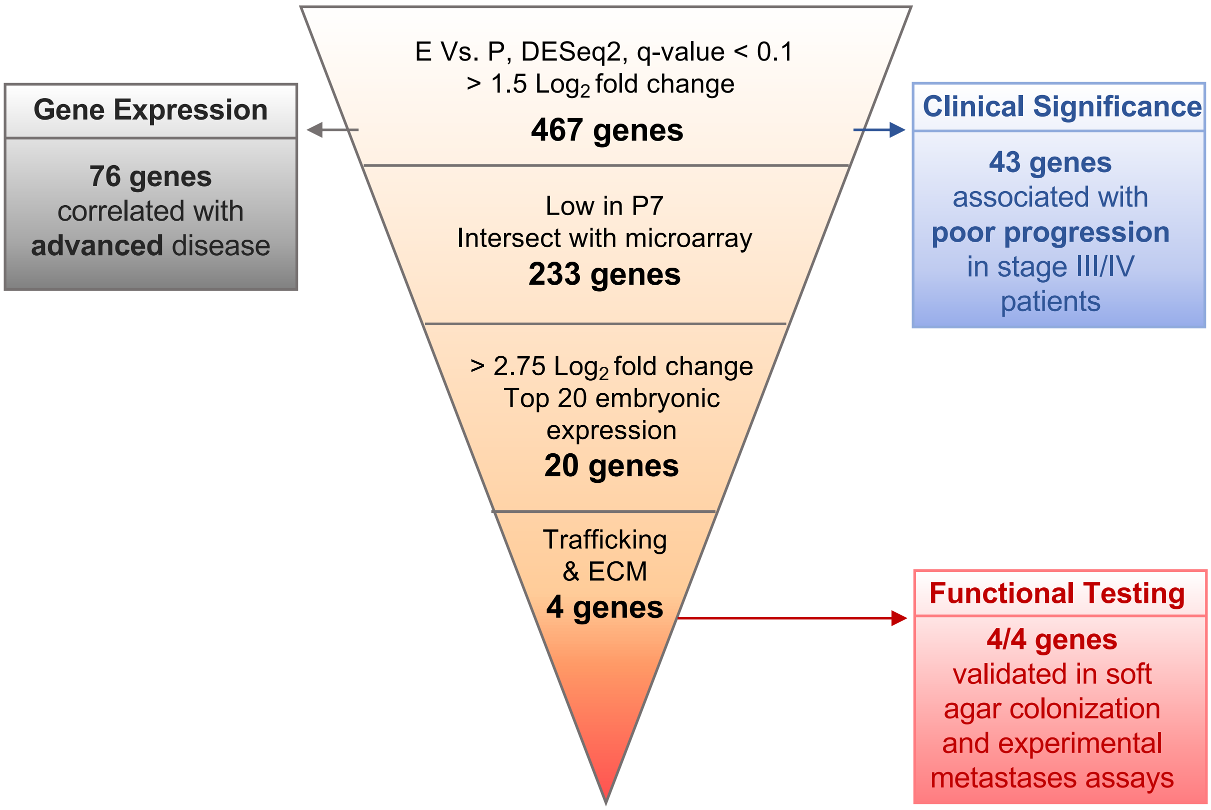

E Vs. P, DESeq2, q-value < 0.1
> 1.5 Log_2 fold change
467 genes
Low in P7
Intersect with microarray
233 genes
> 2.75 Log_2 fold change
Top 20 embryonic expression
20 genes
Trafficking & ECM
4 genes
Gene Expression
76 genes correlated with advanced disease
Clinical Significance
43 genes associated with poor progression in stage III/IV patients
Functional Testing
4/4 genes validated in soft agar colonization and experimental metastases assays

Supplementary Figure 2: Validation of melanoblast genes in metastasis: workflow for gene prioritization. a, Gene expression from Embryonic stages day 15.5 and 17.5 (E) was contrasted with gene expression from Postnatal stages day 1 and 7 (P) using DESeq2, with a False Discovery Rate (FDR) threshold of 0.1, q-value < 0.1 and >1.5 Log_2-fold change to yield 467 genes. Testing gene expression correlation with advanced disease (grey box): 76 genes were upregulated in metastatic melanoma versus primary tumor (GSE8401) and 66 genes with available expression data were validated in GSE98394. Testing clinical significance of MetDev genes (blue box): Cox proportional hazards modeling of these 467 genes in training dataset GSE19234, yielded a 43-gene signature that was associated with poor progression in late-stage (stage III/IV) metastatic melanoma of both GSE19234 and GSE8401 (testing dataset). The 43-gene signature did not predict poor patient prognosis from early stage (stage I/II) primary tumors (GSE8401). Further prioritization of the 467 melanoblast genes took place purely on a statistical basis: All genes with a FPKM > 2 in P7 were filtered out. The intersect of embryonic genes to those determined in a separate microarray study of melanocyte development (E17.5 versus P2 and P7) using a linear regression model, selecting for q-value < 0.1, was determined. 233 genes were prioritized from the 467 gene list. The top 20 genes were determined by > 2.75 Log_2-fold change, P-value < 0.0003, and top 20 highest mean expression from embryonic stages. 4 genes were selected based on their role in trafficking and Extracellular Matrix (ECM), and their novelty in melanoma metastasis.

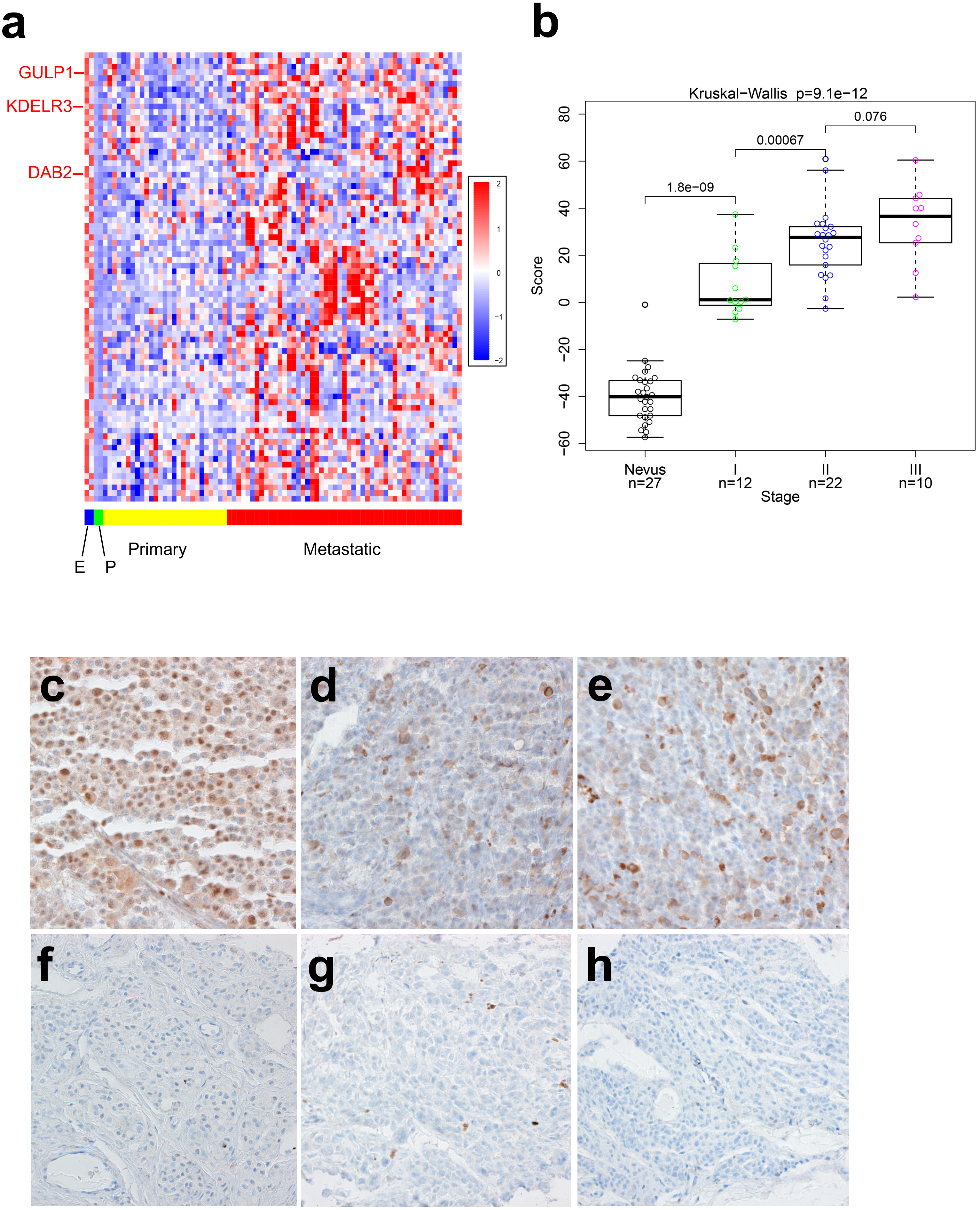
a
GULP1
KDELR3
DAB2
2
1
0
−1
−2
E
P
Primary
Metastatic
b
Kruskal−Wallis p=9.1e−12
Score
80
60
40
20
0
−20
−40
−60
1.8e−09
0.00067
0.076
Nevus
n=27
I
n=12
II
n=22
III
n=10
Stage
c
d
e
f
g
h

Supplementary Figure 3: Increased MetDev gene expression correlates with more advanced melanoma stage . a, Heatmap of expression of 76 embryonic-specific genes in melanoma patients (GSE8401). Primary tumor samples, stage I /II, N = 27; Stages I/II) Metastatic samples, stages III/IV, N = 47. Functionally validated genes indicated. Color scale represents gene expression z-scores. **b**, Computation of a gene expression score of 66/76 available embryonic-specific genes in melanoma patient samples (GSE98394). Kruskal-Wallis test revealed different score distributions among the 4 groups (Nevus and Stages I, II, III), $P = 9.1e-12$. Scores increased across the 4 groups, rho=0.87 and $P = 2.2e-16$. The Wilcoxon test was used to compare paired groups to compute p-values. Boxplots depicting the 25th, 50th (median), 75th percentile, and extreme values of the transcript expression. **c**-**h**, The NCI melanoma progression Tumor Microarray (TMA) panel was stained with anti-KDELR3 (**c**, **f**), anti-P4HA2 (**d**, **g**) and anti-DAB2 (**e**, **h**) antibodies. Metastatic melanoma (visceral) patient samples, N=56 (**c**-**e**) and cutaneous nevi, N=35 (**f**-**h**) were tested. Representative images shown: none of the nevi demonstrated greater than 10% staining, while an appreciable number of the metastatic lesions were positive. All images 200x total magnification, 20x objective magnification.

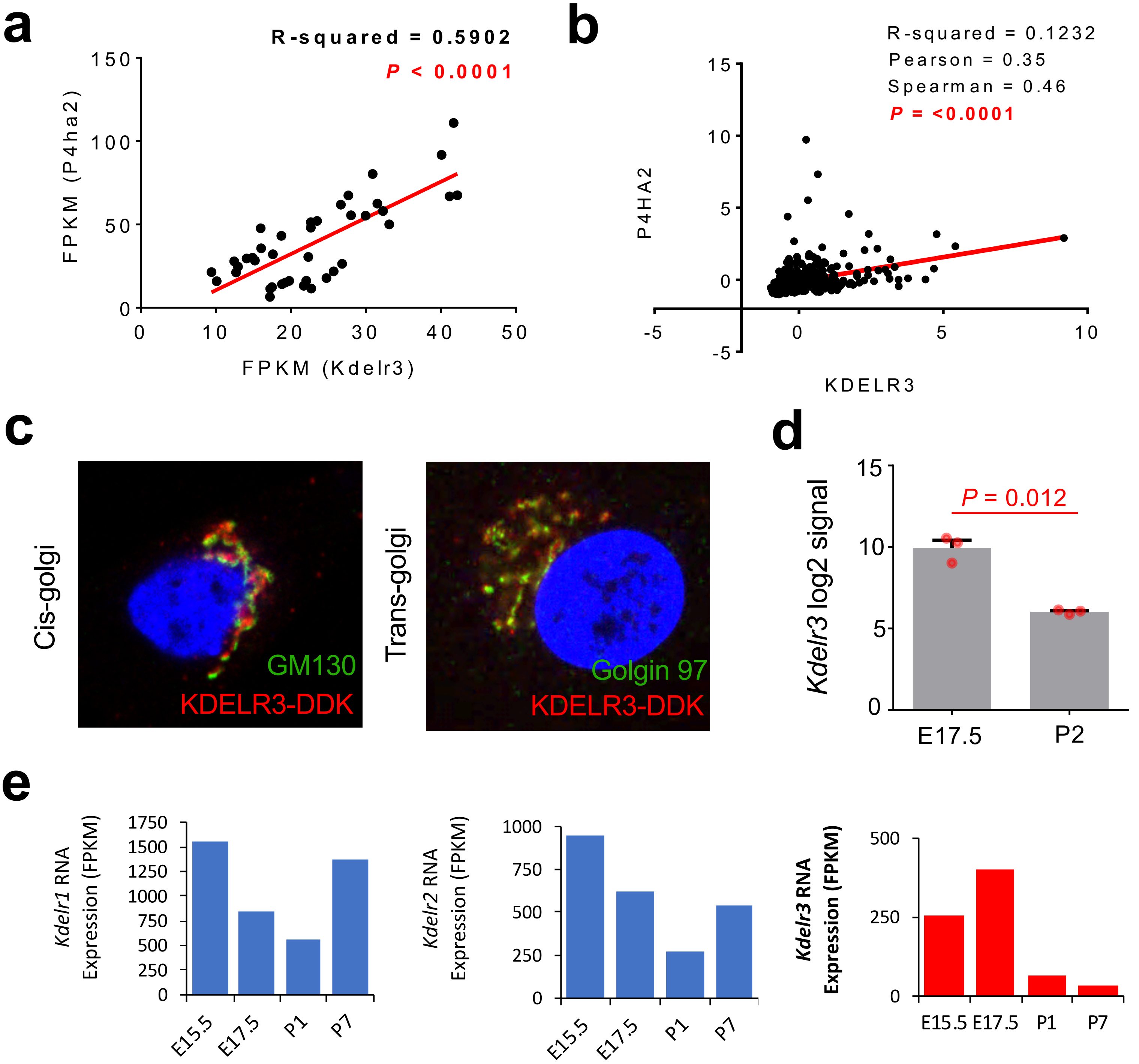

a
R-squared = 0.5902
P < 0.0001
FPKM (P4ha2)
FPKM (Kdelr3)
b
R-squared = 0.1232
Pearson = 0.35
Spearman = 0.46
P = <0.0001
P4HA2
KDELR3
c
Cis-golgi
GM130
KDELR3-DDK
Trans-golgi
Golgin 97
KDELR3-DDK
d
P = 0.012
Kdelr3 log2 signal
E17.5
P2
e
Kdelr1 RNA Expression (FPKM)
Kdelr2 RNA Expression (FPKM)
Kdelr3 RNA Expression (FPKM)
E15.5
E17.5
P1
P7

Supplementary Figure 4: KDELR3 is an ER protein upregulated in melanoma. a, Scatter plot of *P4ha2* RNA expression versus *Kdelr3* RNA expression in four independent mouse models of melanoma. Each dot represents one mouse. M1, *N*= 9 mice; M2, *N*= 6 mice; M3, *N*= 12 mice; M4, *N*= 13 mice. Linear regression analysis, R-squared = 0.5902, $P < 0.0001$. **b**, Scatter plot of *P4ha2* RNA expression versus *Kdelr3* RNA expression in human melanoma patients (cBioPortal, TCGA). Linear regression analysis, R-squared = 0.1232, $P < 0.0001$. Spearman = 0.46. Pearson = 0.35. **c**, Immunofluoresence in the 1205Lu human metastatic melanoma cell line. Flag-tagged KDELR3 expression (red) co-localizes with the cis-golgi marker, GM130 (green; left-hand panel) and trans-golgi maker, Golgin 97 (green, right-hand panel). Representative image of over 50 cells analyzed in three independent experiments. **d**, Independent study showing *Kdelr3* microarray expression data in mouse developing melanocytes[8], $P = 0.012$ n = 3, $t = 8.256$, $df = 2.12$. Unpaired two-tailed t-test with Welch's correction. Bars and error bars depict mean + s.e.m. **e**, RNA-seq data showing *Kdelr* family expression in mouse developing melanocytes**.**

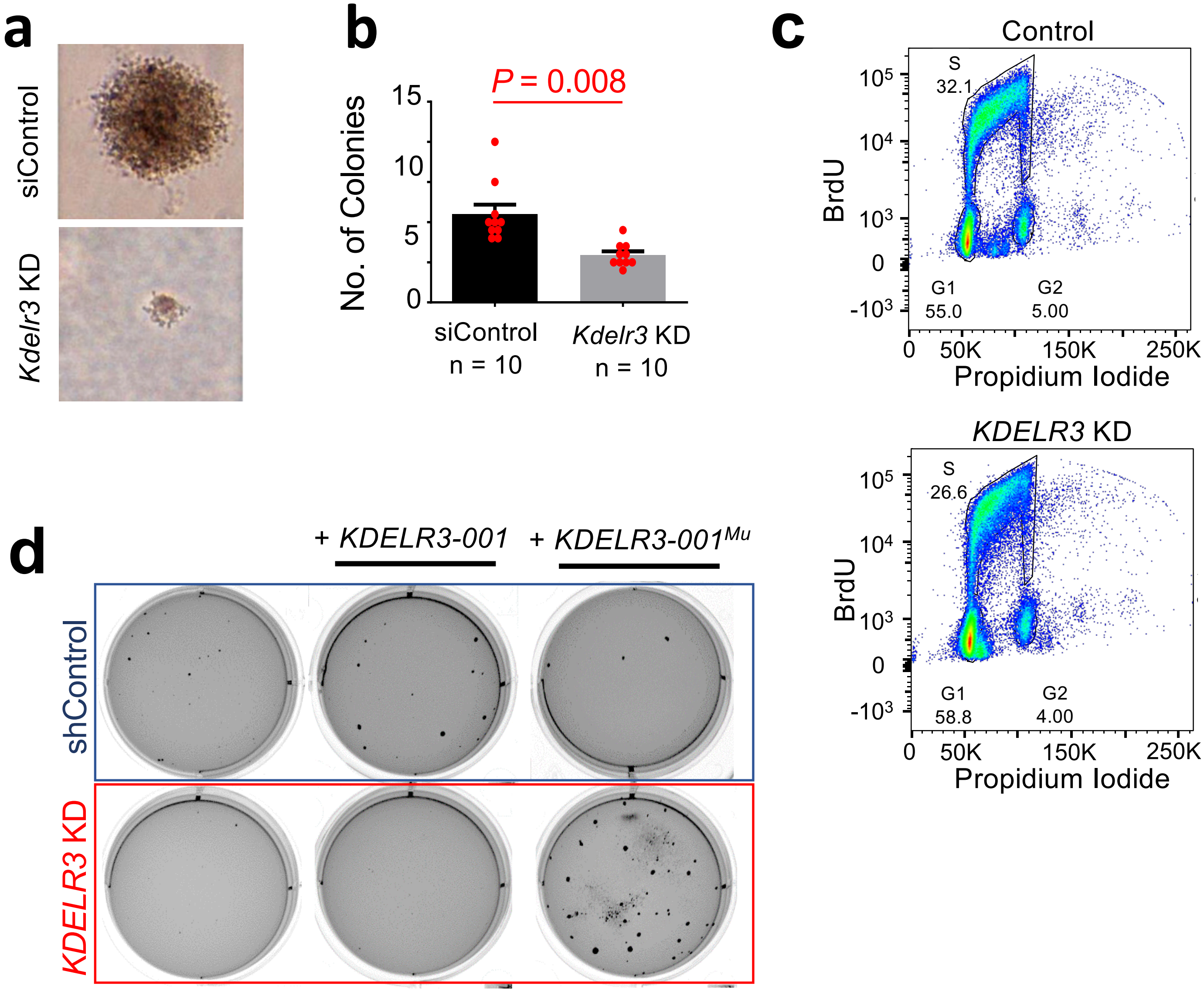

Supplementary Figure 5: *KDELR3* mediates anchorage-independent growth in melanoma. a-b, Soft agar colony formation assay with *Kdelr3* siRNA knockdown in mouse B16 cells versus non-targeting control, unpaired two-tailed student's t-test, P = 0.008, df = 18, t =4.019. 10 wells analyzed per group. Bars and error bars depict mean + s.e.m. **c**, Flow cytometry analysis of BRDU incorporation (Alexa Fluor 488-A) and PI staining (DsRed-A) in WM-46 human melanoma cells. shRNA *KDELR3* knockdown does not affect the cell cycle, when compared to non-targeting control cells. G1-arrest, S-phase and G2-arrest populations were: 55 %, 32.1 %, and 5% in control cells, versus, 58.8 %, 26.8% and 4% in *KDELR3* knockdown cells. **d**, Rescue of soft agar colony formation in *KDELR3-001*^{Mu} cells (WM-46).

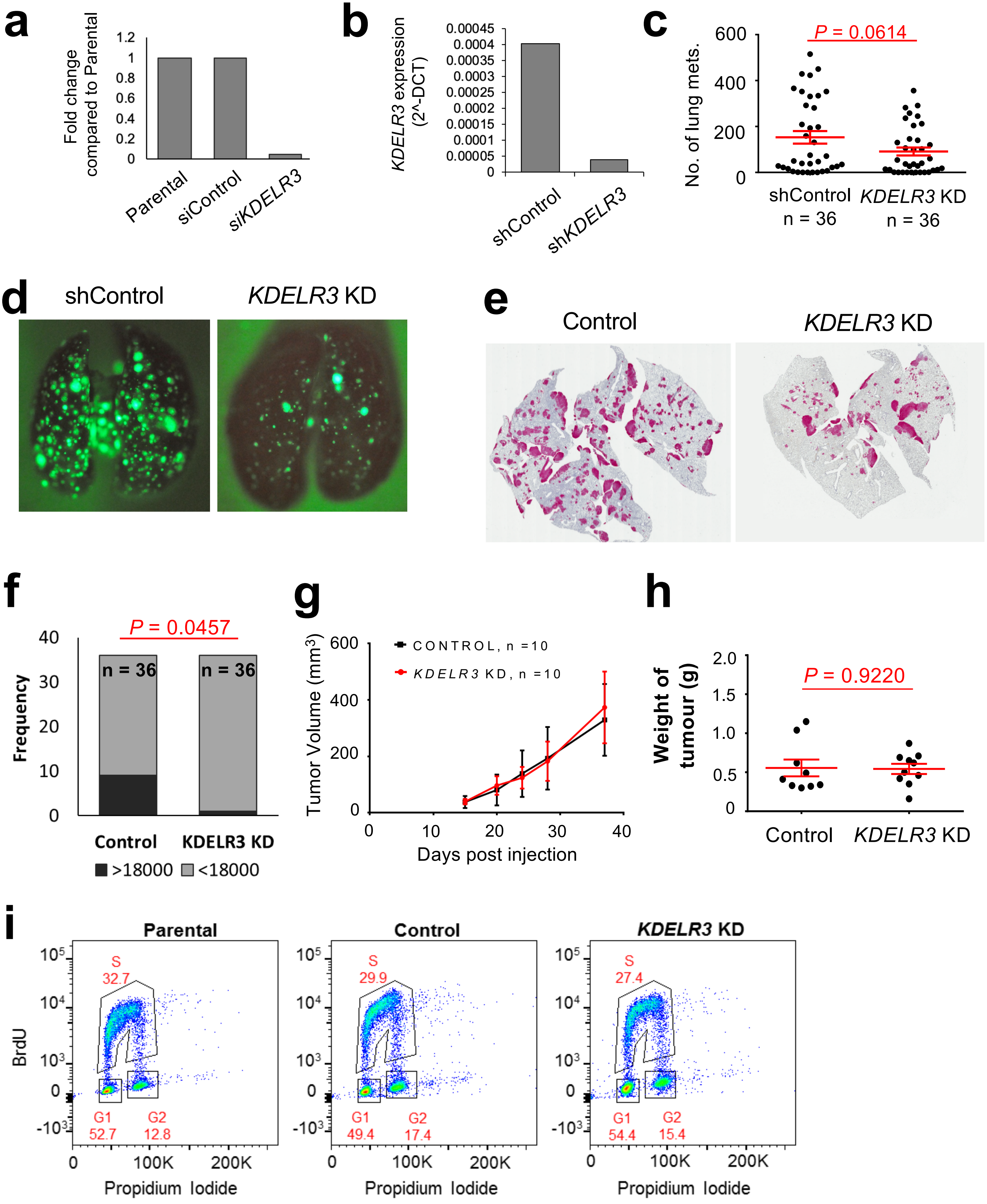
a
Fold change compared to Parental
1.2
1
0.8
0.6
0.4
0.2
0
Parental
siControl
siKDELR3
b
KDELR3 expression (2^-DCT)
0.00045
0.0004
0.00035
0.0003
0.00025
0.0002
0.00015
0.0001
0.00005
0
shControl
shKDELR3
c
P = 0.0614
No. of lung mets.
600
400
200
0
shControl
n = 36
KDELR3 KD
n = 36
d
shControl
KDELR3 KD
e
Control
KDELR3 KD
f
P = 0.0457
Frequency
40
30
20
10
0
n = 36
n = 36
Control
KDELR3 KD
>18000
<18000
g
Tumor Volume (mm3)
600
400
200
0
CONTROL, n =10
KDELR3 KD, n =10
0
10
20
30
40
Days post injection
h
Weight of tumour (g)
2.0
1.5
1.0
0.5
0
P = 0.9220
Control
KDELR3 KD
i
Parental
Control
KDELR3 KD
BrdU
10⁵
10⁴
10³
0
-10³
S
32.7
G1
52.7
G2
12.8
S
29.9
G1
49.4
G2
17.4
S
27.4
G1
54.4
G2
15.4
0
100K
200K
Propidium Iodide

Supplementary Figure 6: *KDELR3* expression in melanoma cells enhances metastatic potential without affecting proliferation. **a**, qPCR data of *KDELR3* expression in 1205Lu cells 3 days following siRNA knockdown of *KDELR3* (si*KDELR3*) or non-targeting control (siControl), as compared to Parental. **b**, qPCR data of *KDELR3* expression following shRNA knockdown of *KDELR3* (sh*KDELR3*) versus non-targeting control (shControl). **c**-**e**, Tail vein metastasis of *KDELR3* shRNA-mediated knockdown human 1205Lu cells transduced with *Ferh-luc-GFP*, (**c**) quantification of numbers of lung metastases. Unpaired two-tailed t-test with Welch's correction, $P = 0.0614$, $df = 59.21$, $t = 1.907$. **d**, Tail vein injection of *Ferh-luc-GFP*-transduced human 1205Lu cells enables visualization of metastases (4 weeks post-injection). **e**, Human-specific HLA-A staining in FFPE sections (n = 36 per group); representative sections shown. **f**, Quantification of total area of metastases per section, determined by arbitrary units (ImageJ), two-tailed Fisher's Exact test, n = 36 lungs. **g**, Effect of *KDELR3* shRNA knockdown (*KDELR3* KD)on subcutaneous tumor growth curve in 1205Lu cells, center and error bars represent mean ± standard deviation. N = 10 mice per group. Representative results from two independent experiments. **h**, Effect of *KDELR3* shRNA knockdown on subcutaneous tumor growth (1205Lu) represented by end tumor weight (40-days post injection). Representative of two independent experiments. **i**, Flow cytometry analysis of BrdU (Alexa Fluor-647) incorporation and Propidium Iodide staining in 1205Lu metastatic melanoma cells. shRNA *KDELR3* knockdown (*KDELR3* KD) does not affect the cell cycle, when compared to non-targeting control (Control) cells or parental cells. G1-arrest, S-phase and G2-arrest populations were: 52.7%, 32.7%, and 12.8% in parental cells; 49.4%, 29.9%, and 17.4% in non-targeting control (Control) cells; 54.4%, 27.4% and 15.4% in *KDELR3* knockdown cells. Representative of two independent experiments carried out in two different cell lines. **c-h**, Non-targeting shRNA control used (Control). **d-f,** Cumulative data taken from three independent experiments. **c**, **h**, Lines and error bars depict mean ± s.e.m. **a-b**, Representative of three independent experiments.

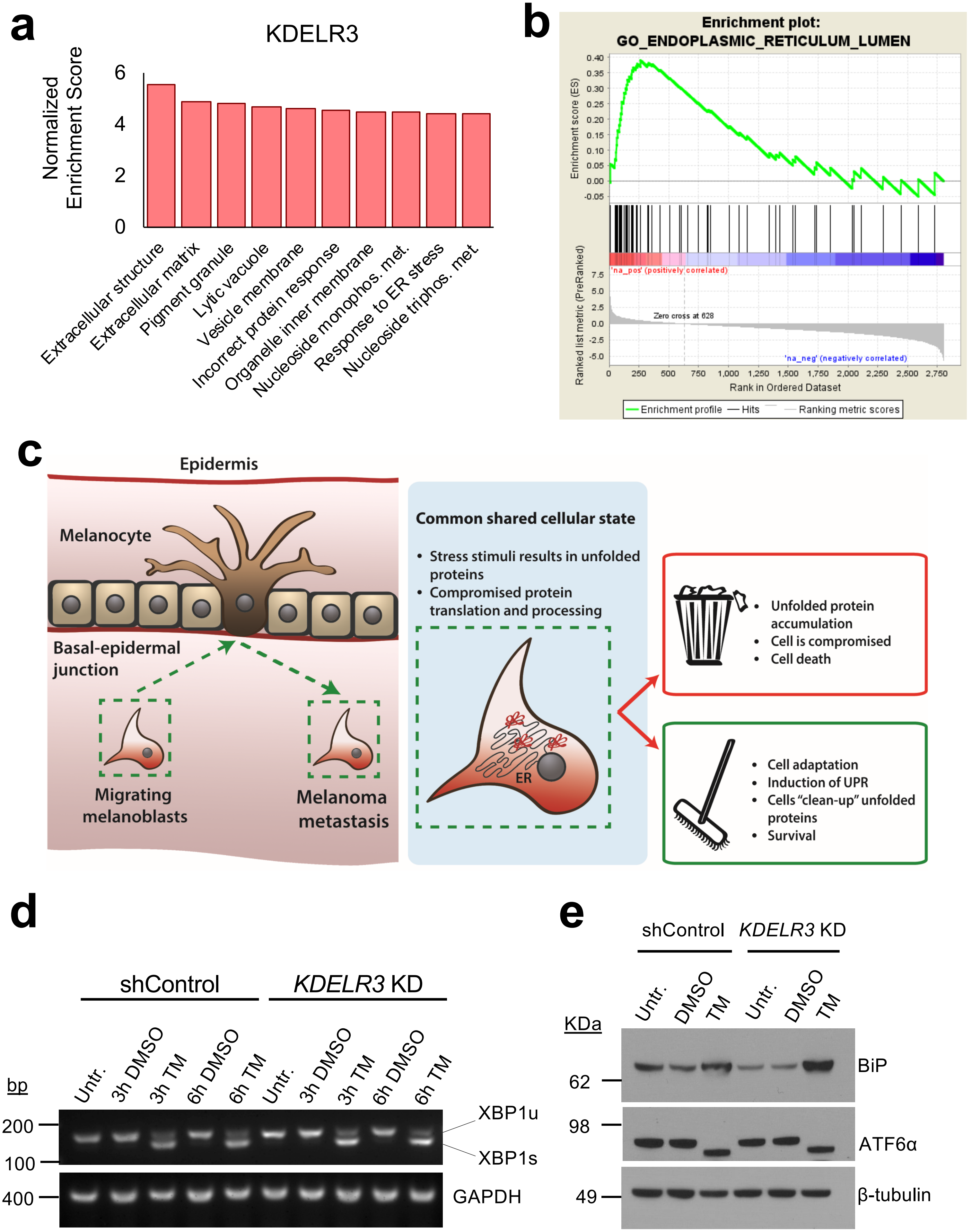
a
KDELR3
Normalized Enrichment Score
6
4
2
0
Extracellular structure
Extracellular matrix
Pigment granule
Lytic vacuole
Vesicle membrane
Incorrect protein response
Organelle inner membrane
Nucleoside monophos. met.
Response to ER stress
Nucleoside triphos. met.
b
Enrichment plot:
GO_ENDOPLASMIC_RETICULUM_LUMEN
Enrichment score (ES)
'na_pos' (positively correlated)
Zero cross at 628
'na_neg' (negatively correlated)
Ranked list metric (PreRanked)
Rank in Ordered Dataset
Enrichment profile — Hits — Ranking metric scores
c
Epidermis
Melanocyte
Basal-epidermal junction
Migrating melanoblasts
Melanoma metastasis
Common shared cellular state
Stress stimuli results in unfolded proteins
Compromised protein translation and processing
ER
Unfolded protein accumulation
Cell is compromised
Cell death
Cell adaptation
Induction of UPR
Cells "clean-up" unfolded proteins
Survival
d
shControl
KDELR3 KD
bp
Untr.
3h DMSO
3h TM
6h DMSO
6h TM
200
100
400
XBP1u
XBP1s
GAPDH
e
shControl
KDELR3 KD
Untr.
DMSO
TM
KDa
62
98
49
BiP
ATF6α
β-tubulin

Supplementary Figure 7: *KDELR3* and the ER Stress Response in metastatic melanoma. a, GSEA of gene co-expression within skin cutaneous melanoma patients of the TCGA (n = 479). Top 10 *KDELR3*-associated GO pathways represented, FDR < 0.0001. Go pathways in order: Extracellular structure organization; Extracellular matrix; Pigment granule; Lytic vacuole; Vesicle membrane; Response to topologically incorrect protein; Organelle inner membrane; Nucleoside monophosphate metabolic process; Response to endoplasmic reticulum stress; Nucleoside triphosphate metabolic process. **b**, Gene Ontology (GO) term enrichment of positively enriched pathways following proteomic analysis of si*KDELR3* knockdown cells (1205Lu) compared to parental cells and non-targeting control cells identified GO_ENDOPLASMIC_RETICULUM_LUMEN as the GeneSet consistently enriched in two independent replicates. Enrichment Score (ES) = 0.39025578, Normalized Enrichment Score (NES) = 1.823677, Nominal p-value < 0.0001, FDR q-value = 0.09752231, FWER p-value = 0.186. Representative results from two independent experiments. **c**, Migrating melanoblasts and metastasizing melanoma cells are affected by similar stress stimuli, i.e. low oxygen/nutrient supply, foreign microenvironment, detachment, shear stressors. Sub-optimal protein translation and processing during times of cellular stress results in build-up of unfolded proteins in the ER-Golgi transport network (ER stress). Unfolded protein accumulation further compromises normal protein translation, processing and transport, thereby affecting cellular function. Inability to cope with ER stress typically results in cell death. Induction of the unfolded protein response (UPR) is a cell survival mechanism that allows cells to adapt to ER stress. **d**, RT-PCR analysis of XBP1 splicing in shRNA non-targeting control and sh*KDELR3* knockdown (1205Lu cells). Un-spliced, inactive XBP1 (XBP1u) was compared to spliced, active XBP1 signaling (XBP1s) Cells were treated for the time specified with: untreated, DMSO, or 3μg/ml Tunicamycin (TM). GAPDH loading control. Representative results from two independent experiments in two different cell lines (WM-46 and 1205Lu)**. e**, BiP and ATF6α protein expression in non-targeting control (shControl) and sh*KDELR3* (*KDELR3* KD) WM-46 cells in untreated (Untr.), DMSO controls (DMSO) and treated with 2.5 μg/ml Tunicamycin (TM) 15 hours before collection. BiP: representative results from three independent cell lines (1205Lu, WM-46, B16). ATF6α: representative results from three independent experiments in two cell lines (WM-46 and 1205Lu).

a

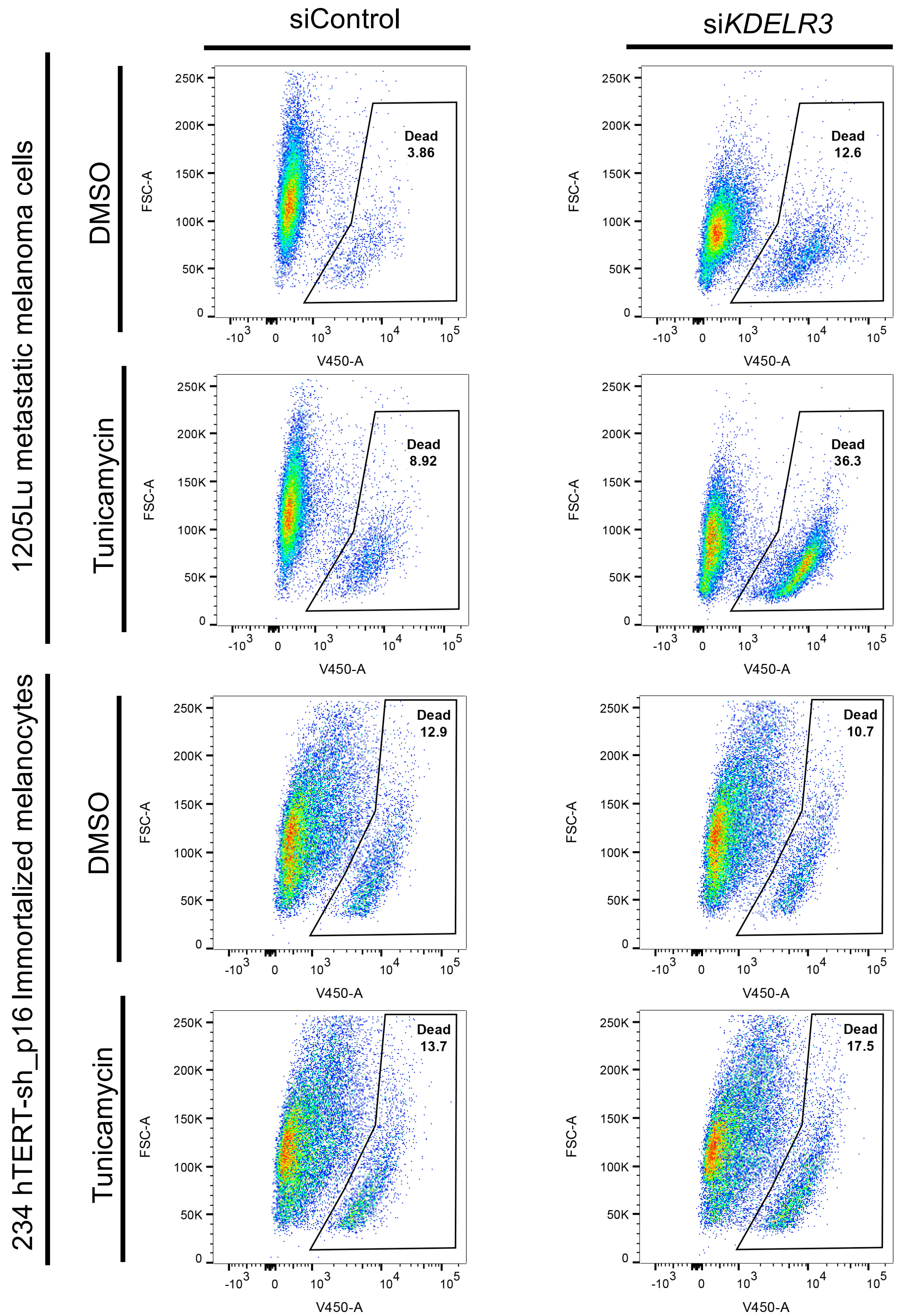

siControl
siKDELR3
1205Lu metastatic melanoma cells
234 hTERT-sh_p16 Immortalized melanocytes
DMSO
Tunicamycin
DMSO
Tunicamycin
FSC-A
V450-A
Dead 3.86
Dead 12.6
Dead 8.92
Dead 36.3
Dead 12.9
Dead 10.7
Dead 13.7
Dead 17.5

b

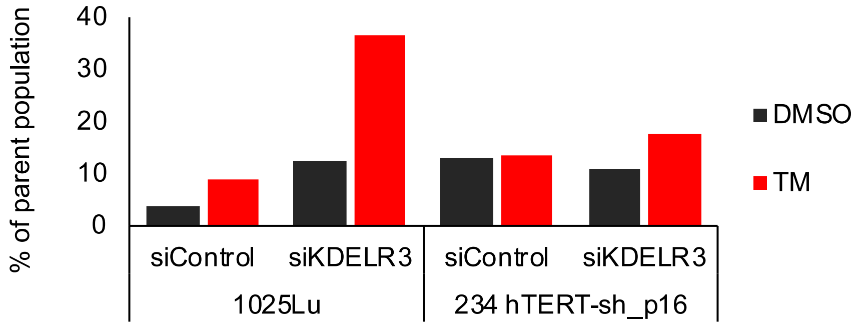

% of parent population
40
30
20
10
0
siControl
siKDELR3
siControl
siKDELR3
1025Lu
234 hTERT-sh_p16
DMSO
TM

Supplementary Figure 8: *KDELR3* is critical for metastatic melanoma cell viability, but not for melanocyte viability. a-b, Live/dead violet cell stain in *KDELR3*-knockdown 1205Lu metastatic melanoma cells and 234. hTERT-sh_p16 immortalized melanocytes. DMSO, and tunicamycin (2.5 μg/ml) treatment groups were treated 23 hours before collection. Percentage dead cells of the parent population are indicated. Representative of 3 independent experiments.

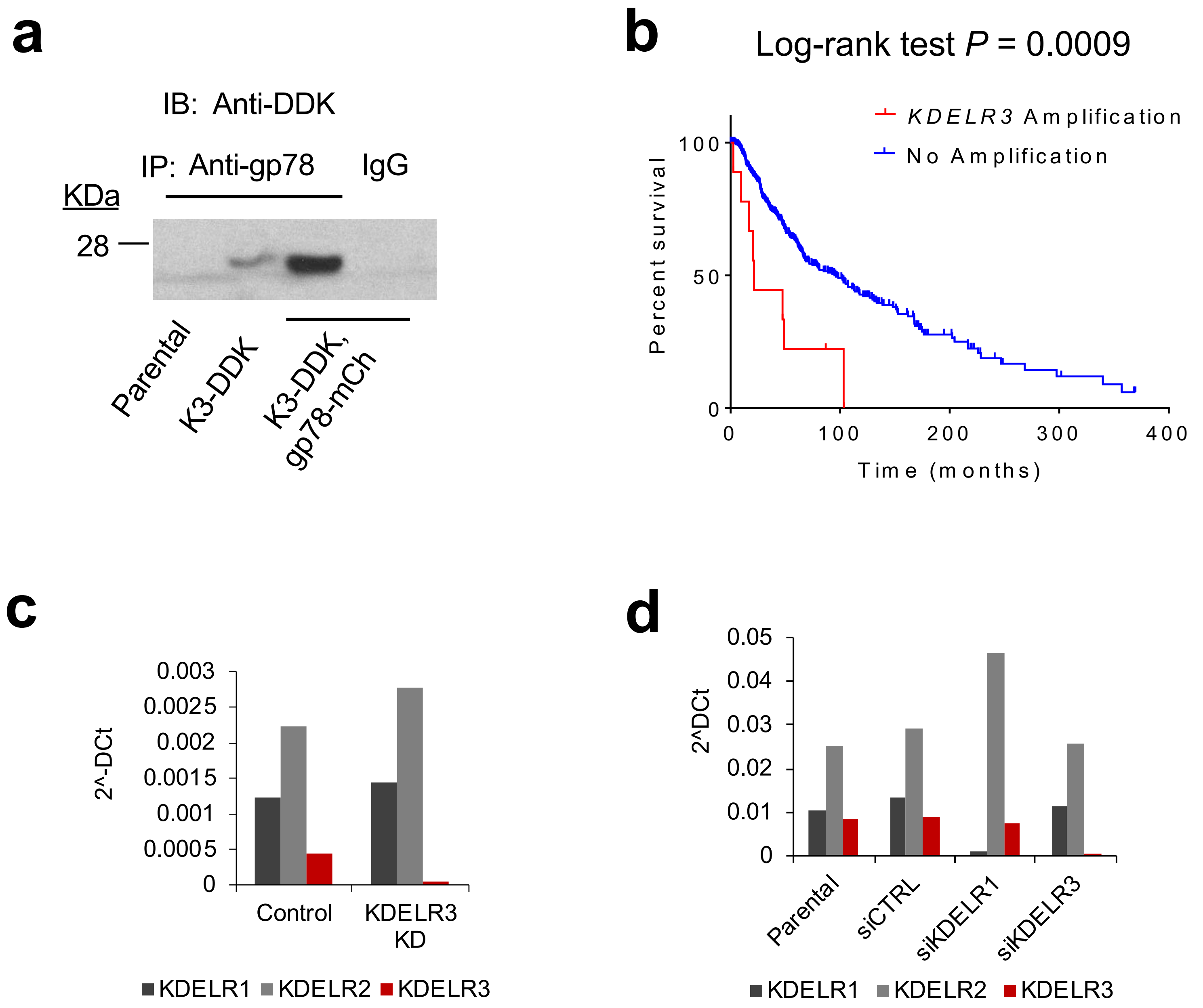

Supplementary Figure 9: KDELR3 biology in melanoma. a, Co-immunoprecipitation of endogenous gp78 and mCherry tagged gp78 (gp78-mCh) with FLAG-tagged KDELR3 (K3-DDK) in stably transduced 1205Lu cells. **b**, Kaplan-Meier curve of skin cutaneous melanoma patient (TCGA-SKCM) overall survival with (N=9) and without (N=349) *KDELR3* CNV amplifications; CNV determined by GISTIC 2.0. Data generated by cBioPortal for Cancer Genomics. Log-rank test, P = 0.009. **c-d**, KDELR family member expression in 1205Lu metastatic melanoma cells following shRNA (**c**) or siRNA knockdown (**d**; day 3 post knockdown). **a**, **c-d**, Representative of three independent experiments.

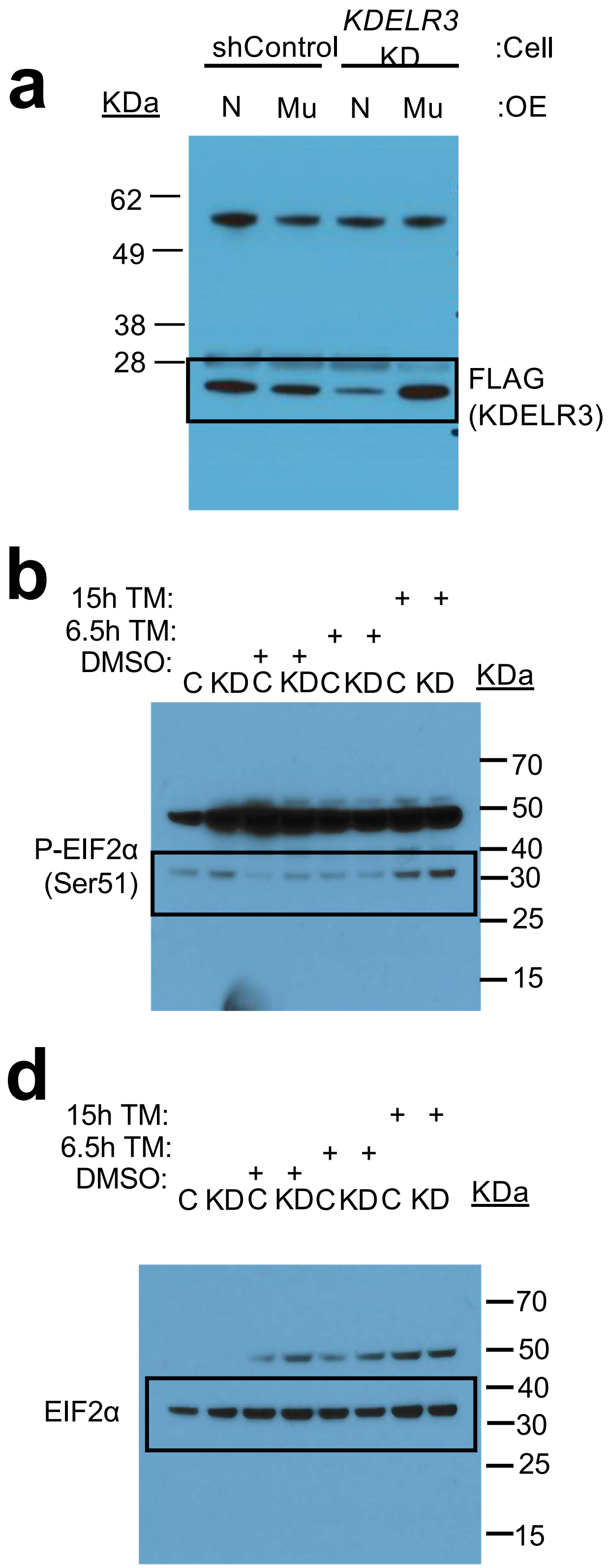

Supplementary Figure 10: Uncropped/unprocessed films of all most important blots. Blots correspond to Figure 2a (a), Figure 4c (b-e). Black open box represents cropped region in figures. **a**, Western blot of exogenously expressed FLAG-tagged *KDELR3-001*; ENST00000216014 (N) and *KDELR3-001*^{Mu} (Mu) in WM-46 cells. **b-e**, Western blot analysis of PERK and eIF2α signaling (1205Lu cells). Non-targeting control (shControl; C) and *KDELR3* knockdown (sh*KDELR3*; KD) cells were untreated or treated with 3 μg/ml tunicamycin (TM) for the indicated time. Immunoblot with antibodies specified, β-Tubulin loading control.

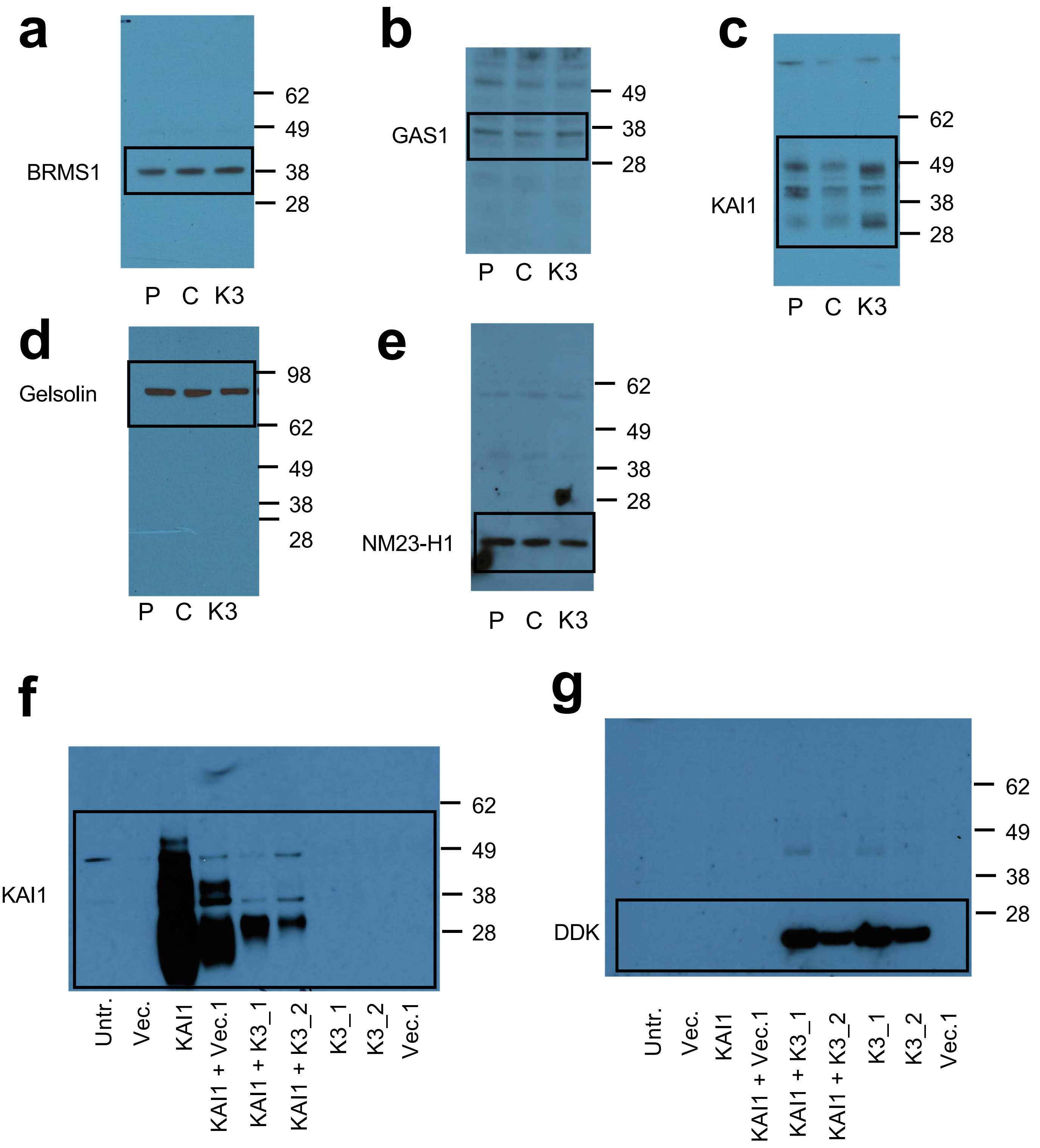

Supplementary Figure 11: Uncropped/unprocessed films of all most important blots. Blots correspond to Figure 5a (a-e), Figure 5c (f-g). Black open box represents cropped region in figures. **a-e**, Screen of known melanoma metastasis suppressor expression following *KDELR3* knockdown (3 days post knockdown). P, Parental; C, siControl; K3, si*KDELR3*. **f-g**, KAI1 protein expression in 1205Lu cells transfected with CD82/KAI1 overexpression (KAI1) or PCMV6-AC control vector (Vec.), *KDELR3* transcript 1with DDK tag (K3_1), *KDELR3* transcript 2 with DDK tag (K3_2), or PCMV6 control vector (Vec.1). Harvested 3 days post transfection.

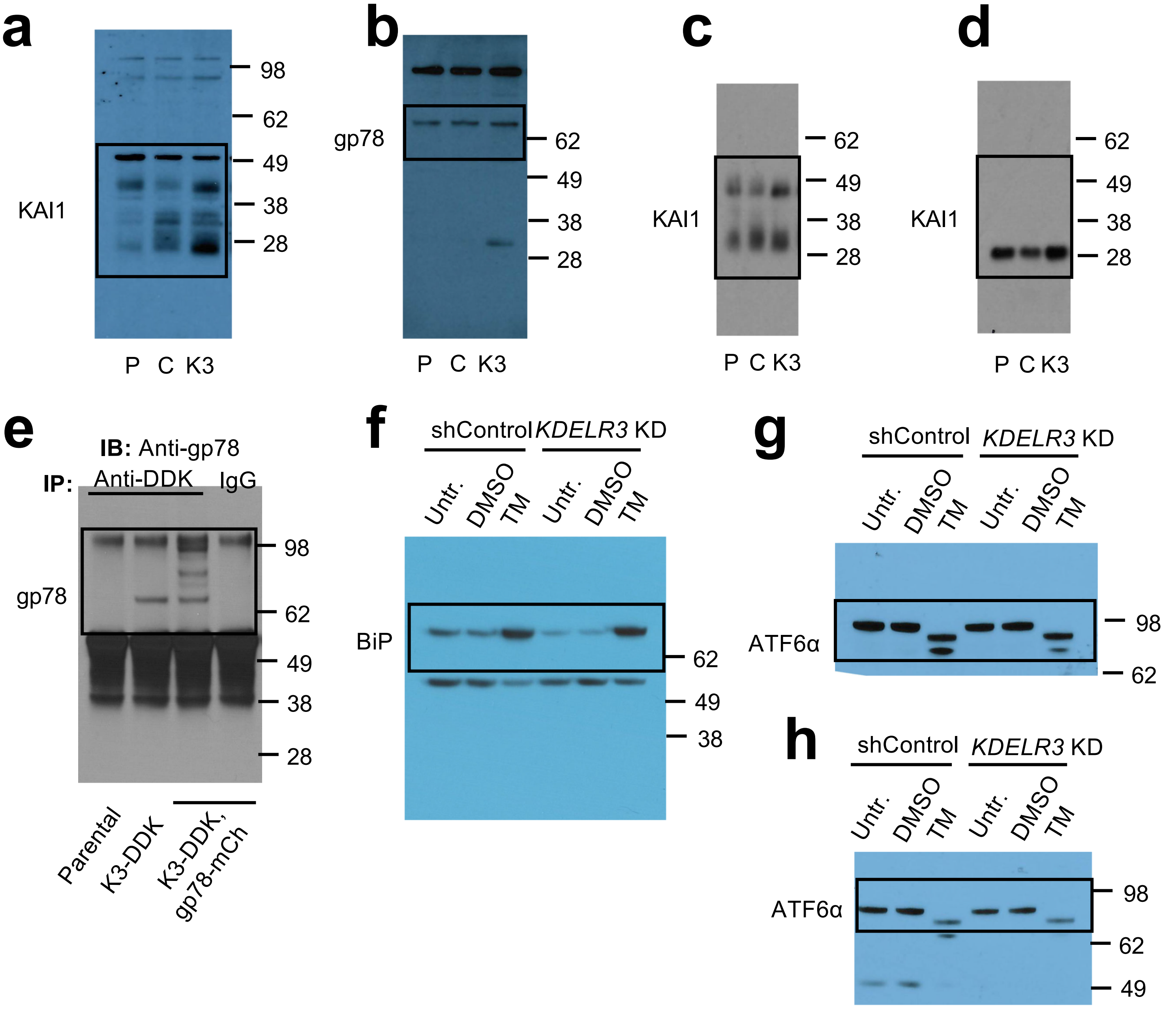

Supplementary Figure 12: Uncropped/unprocessed films of all most important blots. Blots correspond to Figure 5f (a-b), Figure 5g (c-d), Figure 5j (e), Supplementary Figure 7e (f-h). Black open box represents cropped region in figures. **a-b**, 1205Lu cells parental (P), and 1205Lu cells transiently transfected with control siRNA (C), and *KDELR3* siRNA (K3), harvested 3 days post transfection and equal protein amounts subjected to immunoblot analysis with an anti-KAI1 and anti-gp78 antibody. **c-d**, KAI1 protein expression in siRNA knockdown (indicated) 1205Lu cells harvested 3 days post transfection and treated with de-glycosylation enzymes (De-G). **e**, Co-immunoprecipitation of endogenous gp78 and mCherry tagged gp78 (gp78-mCh) with FLAG-tagged KDELR3 (K3-DDK) in stably transduced 1205Lu cells. **f-h**, BiP and ATF6α protein expression in non-targeting control (shControl) and sh*KDELR3* (*KDELR3* KD) WM-46 cells in untreated (Untr.), DMSO controls (DMSO) and treated with 2.5 μg/ml Tunicamycin (TM) 15 hours before collection.

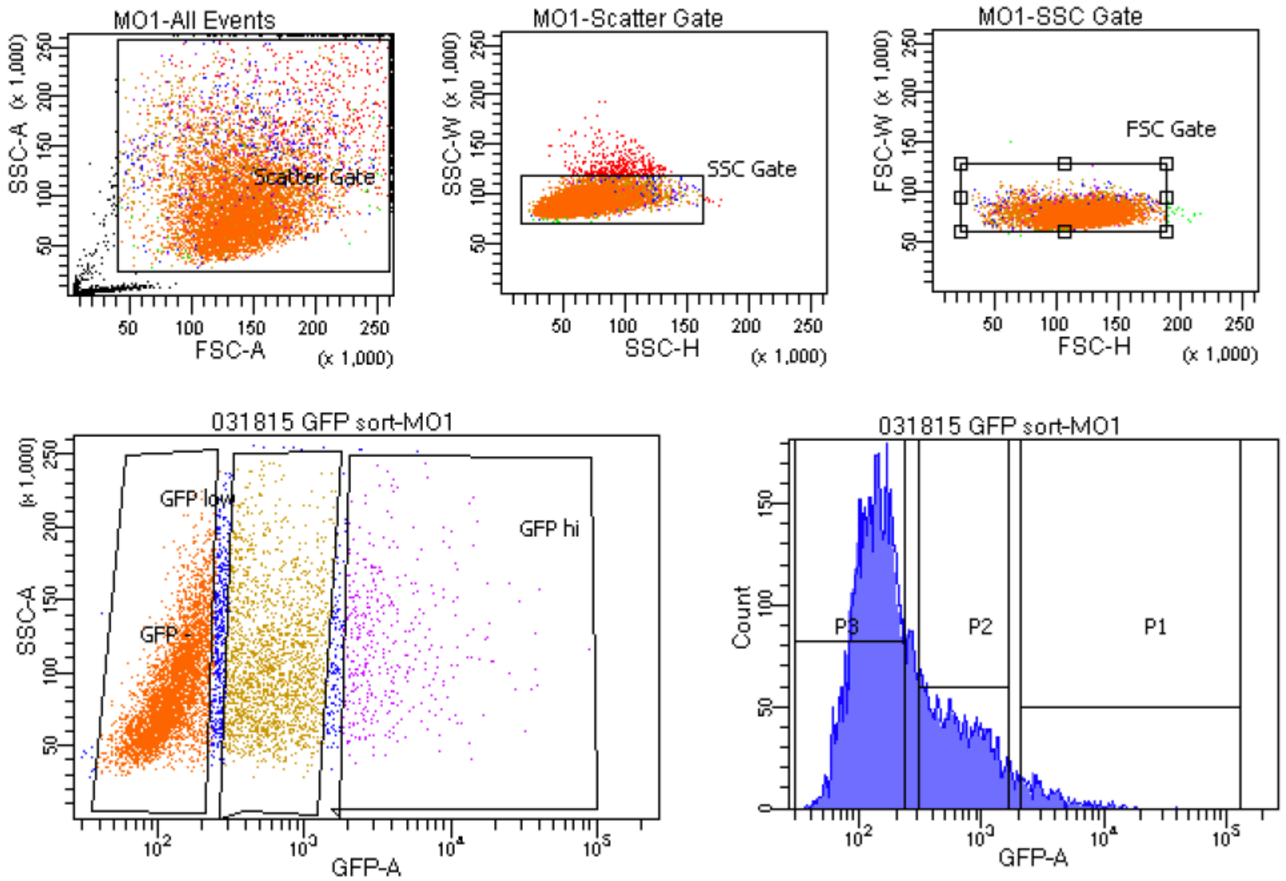

Tube: MO1

Population	#Events	%Parent	%Total
All Events	10,000	####	100.0
Scatter Gate	7,933	79.3	79.3
SSC Gate	7,536	95.0	75.4
FSC Gate	7,500	99.5	75.0
GFP hi	394	5.3	3.9
GFP low	1,924	25.7	19.2
P1	348	4.6	3.5
P2	1,931	25.7	19.3
GFP -	4,569	60.9	45.7
P3	4,552	60.7	45.5

Supplementary Figure 13: FACS sorting of mPol2p> Kz-KDELR3-eGFP IRES>ffluc2 transduced cells. Representative gating strategy for cell sorting.

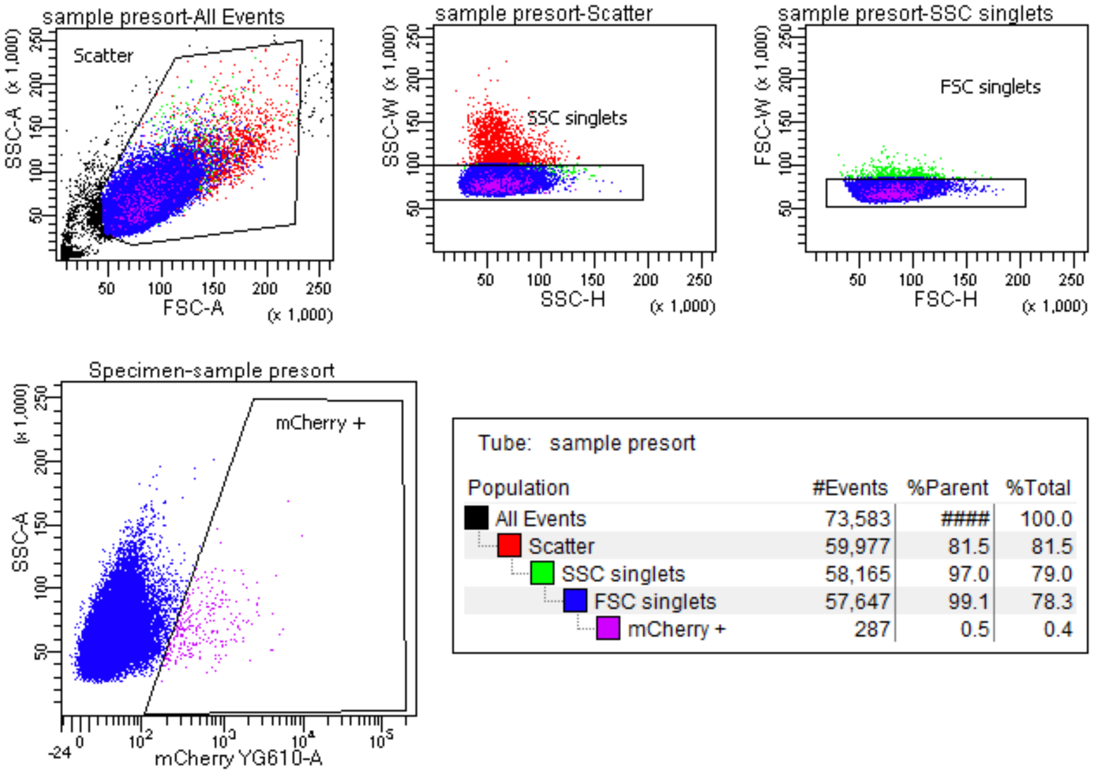

Population	#Events	%Parent	%Total
All Events	73,583	####	100.0
Scatter	59,977	81.5	81.5
SSC singlets	58,165	97.0	79.0
FSC singlets	57,647	99.1	78.3
mCherry +	287	0.5	0.4

Supplementary Figure 14: FACS sorting of mPol2p>Hs.AMFR-mCherry transduced cells. Representative gating strategy for cell sorting.

Gene comparison	R-squared	1/slope	P-value	Significance?
Kdelr3 vs P4ha2	0.5902	0.4608	< 0.0001	Yes
Kdelr3 vs Gulp1	0.02636	-5.53	0.3169	No
Kdelr3 vs Dab2	0.4415	0.6481	< 0.0001	Yes
P4ha2 vs Gulp1	0.02643	15.6	0.3162	No
P4ha2 vs Dab2	0.3827	1.966	< 0.0001	Yes
Gulp1 vs Dab2	0.00099	-15.26	0.8473	No

Supplementary Table 1: Co-expression of 4 functionally validated genes within four independent mouse melanoma models. Significance assessed by linear regression analysis.

Gene Comparison	Spearman's Correlation	p-Value	q-Value	Significance?
KDELR3 vs P4HA2	0.435	3.49e-23	4.40e-20	Yes
KDELR3 vs DAB2	0.213	2.999e-6	4.169e-5	Yes
KDELR3 vs GULP1	-0.0201	0.662	0.774	No
P4HA2 vs GULP1	-0.00202	0.965	0.978	No
P4HA2 vs DAB2	0.173	1.614e-4	8.925e-4	Yes
GULP1 vs DAB2	-0.273	0.1.74e-9	8.39e-9	Yes

Supplementary Table 2: Co-expression of 4 functionally validated genes within melanoma patients. TCGA, cBioPortal. P-value derived from two sided t-test.

NAME	SIZE	ES	NES	NOM p-val	FDR q-val	FWER p-val	RANK AT MAX
**GO_ENDOPLASMIC_RETICULUM**	364	0.205073	4.232328	0	0	0	1219
**GO_ENDOPLASMIC_RETICULUM_PART**	282	0.22867	4.218896	0	0	0	1122
**GO_ENDOPLASMIC_RETICULUM_LUMEN**	58	0.466685	4.165108	0	0	0	1094
**GO_NUCLEAR_OUTER_MEMBRANE_ENDOPLASMIC_RETICULUM_MEMBRANE_NETWORK**	242	0.178756	3.147752	0	0	0	1122
**GO_EXTRACELLULAR_STRUCTURE_ORGANIZATION**	56	0.330455	2.896429	0	6.30E-04	0.004	1006
GO_VESICLE_COATING	37	0.399024	2.860223	0	7.80E-04	0.006	1053
**GO_GOLGI_APPARATUS_PART**	155	0.188323	2.639097	0	0.006592	0.058	1053
**GO_GOLGI_MEMBRANE**	133	0.201222	2.582831	0.001934	0.008083	0.081	1046
GO_SINGLE_ORGANISM_MEMBRANE_BUDDING	37	0.346847	2.525803	0	0.009166	0.112	1046
**GO_GOLGI_APPARATUS**	254	0.142598	2.545466	0	0.009326	0.105	1075
GO_TRANSPORT_VESICLE	73	0.249239	2.49897	0	0.010362	0.136	1046
GO_VESICLE_TARGETING	38	0.3275	2.400361	0	0.017508	0.245	1046
**GO_ENDOPLASMIC_RETICULUM_GOLGI_INTERMEDIATE_COMPARTMENT**	41	0.321227	2.40303	0	0.018265	0.236	1055
GO_MEMBRANE_BUDDING	49	0.288477	2.385064	0	0.018397	0.278	1053
GO_PROTEINACEOUS_EXTRACELLULAR_MATRIX	39	0.321062	2.357299	0	0.021642	0.339	945
GO_LOCALIZATION_WITHIN_MEMBRANE	43	0.301722	2.322892	0.001916	0.025717	0.417	893
GO_MITOCHONDRIAL_PART	312	0.116061	2.274941	0	0.033188	0.523	1280
GO_ER_TO_GOLGI_TRANSPORT_VESICLE	25	0.376966	2.246624	0	0.036413	0.594	1043
GO_TRANSPORT_VESICLE_MEMBRANE	27	0.359304	2.249874	0	0.037419	0.583	1139
GO_GLYCOPROTEIN_METABOLIC_PROCESS	51	0.259467	2.224952	0.002041	0.040386	0.637	1218

Supplementary Table 3: Gene Set Enrichment Analysis (GSEA) of positively enriched pathways following si*KDELR3* knockdown compared to non-targeting control cells. Gene Ontology (GO) term enrichment. FDR < 0.05 denotes significant pathways, significance assessed by permutation test. ES, Enrichment Score; NES, Normalized Enrichment Score. Bold font denotes pathways consistent with siAMFR knockdown.

NAME	SIZE	ES	NES	NOM p-val	FDR q-val	FWER p-val	RANK AT MAX
**GO_ENDOPLASMIC_RETICULUM**	364	0.191889	4.097375	0	0	0	1465
**GO_ENDOPLASMIC_RETICULUM_PART**	282	0.213989	3.958402	0	0	0	1033
**GO_NUCLEAR_OUTER_MEMBRANE_ENDOPLASMIC_RETICULUM_MEMBRANE_NETWORK**	242	0.19813	3.406401	0	0	0	1465
**GO_ENDOPLASMIC_RETICULUM_LUMEN**	58	0.309021	2.7944	0	0.002871	0.013	1371
GO_PROTEIN_LOCALIZATION_TO_ENDOPLASMIC_RETICULUM	93	0.222677	2.561967	0	0.009951	0.067	1945
**GO_ENDOPLASMIC_RETICULUM_GOLGI_INTERMEDIATE_COMPARTMENT**	41	0.342655	2.567371	0	0.01106	0.062	998
**GO_EXTRACELLULAR_STRUCTURE_ORGANIZATION**	56	0.283743	2.482635	0	0.018637	0.143	599
GO_PROTEIN_N_LINKED_GLYCOSYLATION	21	0.432285	2.323848	0	0.030658	0.406	1027
**GO_GOLGI_APPARATUS**	254	0.131271	2.32819	0	0.032158	0.404	1443
GO_MULTI_ORGANISM_METABOLIC_PROCESS	103	0.206993	2.401925	0.001996	0.03278	0.265	1900
GO_VESICLE_COAT	24	0.395935	2.297542	0	0.03318	0.475	1415
GO_GOLGI_VESICLE_TRANSPORT	111	0.196268	2.332478	0	0.033759	0.394	1486
GO_REGULATION_OF_ENDOTHELIAL_CELL_PROLIFERATION	15	0.504046	2.368323	0	0.034191	0.323	986
GO_PROTEIN_TARGETING_TO_MEMBRANE	97	0.198248	2.301479	0	0.034262	0.464	1961
**GO_GOLGI_APPARATUS_PART**	155	0.160576	2.338985	0.002004	0.035189	0.385	688
GO_ESTABLISHMENT_OF_PROTEIN_LOCALIZATION_TO_ENDOPLASMIC_RETICULUM	87	0.217569	2.348091	0	0.036006	0.368	1945
GO_POSITIVE_REGULATION_OF_EPITHELIAL_CELL_PROLIFERATION	16	0.486615	2.369748	0	0.037693	0.32	887
**GO_GOLGI_MEMBRANE**	133	0.170127	2.230692	0	0.048454	0.636	1004

Supplementary Table 4: Gene Set Enrichment Analysis (GSEA) of positively enriched pathways following si*AMFR* knockdown compared to non-targeting control cells. Gene Ontology (GO) term enrichment. FDR < 0.05 denotes significant pathways, significance assessed by permutation test. ES, Enrichment Score; NES, Normalized Enrichment Score. Bold font denotes pathways consistent with si*KDELR3* knockdown.
